# *Drosophila* Spd-2 Recruits PCM to the Sperm Centriole, but Is Dispensable for Centriole Duplication

**DOI:** 10.1016/j.cub.2007.08.065

**Published:** 2007-10-23

**Authors:** Carly I. Dix, Jordan W. Raff

**Affiliations:** 1 The Gurdon Institute, Tennis Court Road, Cambridge CB2 1QN, United Kingdom

**Keywords:** CELLBIO, CELLCYCLE

## Abstract

In *C. elegans,* genome-wide screens have identified just five essential centriole-duplication factors: SPD-2, ZYG-1, SAS-5, SAS-6, and SAS-4 [1–8]. These proteins are widely believed to comprise a conserved core duplication module [3, 9–14]. In worm embryos, SPD-2 is the most upstream component of this module, and it is also essential for pericentriolar material (PCM) recruitment to the centrioles [1, 4, 15, 16]. Here, we show that *Drosophila* Spd-2 (DSpd-2) is a component of both the centrioles and the PCM and has a role in recruiting PCM to the centrioles. DSpd-2 appears not, however, to be essential for centriole duplication in somatic cells. Moreover, PCM recruitment in *DSpd-2* mutant somatic cells is only partially compromised, and mitosis appears unperturbed. In contrast, DSpd-2 is essential for proper PCM recruitment to the fertilizing sperm centriole, and hence for microtubule nucleation and pronuclear fusion. DSpd-2 therefore appears to have a particularly important role in recruiting PCM to the sperm centriole. We speculate that the SPD-2 family of proteins might only be absolutely essential for the recruitment of centriole duplication factors and PCM to the centriole(s) that enter the egg with the fertilizing sperm.

## Results and Discussion

### DSpd-2 Is a Centriolar Protein That Is Recruited to the PCM during Mitosis

**Figure 1:**
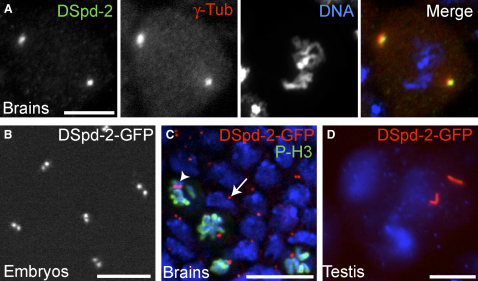
DSpd-2 Localizes to the Centrioles throughout the Cell Cycle and to the PCM in Mitosis (A) WT mitotic third-instar larval neuroblast stained for DSpd-2 (green), γ-tubulin (red), and DNA (blue). DSpd-2 localizes to a broad spot at the mitotic centrosome and largely colocalizes with the PCM marker γ-tubulin. (B) The behavior of DSpd-2-GFP in a syncytial embryo. DSpd-2-GFP is present at the centrosome throughout the embryonic cell cycle (see Movie S1). The embryo shown here is in telophase, and DSpd-2-GFP can be seen on both centrosomes after duplication. (C) Whole-mount third-instar larval brain expressing DSpd-2-GFP (red), stained for Phospho-Histone-H3 (green) to mark mitotic cells and DNA (blue). DSpd-2-GFP localizes to small dots (centrioles) in interphase cells (arrow) and to the PCM in mitotic cells (arrowhead). (D) A larval primary spermatocyte expressing DSpd-2-GFP (red). DNA is also shown (blue). DSpd-2-GFP localizes along the entire length of the two orthogonally arranged pairs of centriole barrels. Scale bars represent 10 μm in (A), (B), and (D) and 5 μm in (C).

The predicted *Drosophila* ortholog of *C. elegans* SPD-2 is encoded by the gene CG17286, which hereafter we refer to as *Drosophila Spd-2* (*DSpd-2*) [1]. We generated and affinity-purified rabbit DSpd-2 antibodies, which recognized centrosomes at all stages of the cell cycle in embryos (data not shown) and specifically in mitosis in larval brain cells (Figure 1A). We also generated a DSpd-2-GFP (green fluorescent protein) fusion protein, which localized to centrosomes throughout the cell cycle in both embryos and larval brain cells (Figures 1B and 1C; Movie S1 in the Supplemental Data available online).

In brain cells, DSpd-2-GFP was more strongly recruited to mitotic centrosomes, but it was also localized to interphase centrosomes (Figure 1C). Because larval brain cells recruit very little pericentriolar material (PCM) during interphase [17], this localization suggests that DSpd-2-GFP is associated with centrioles in interphase and is also recruited to the PCM in mitosis. In addition, we found that DSpd-2-GFP localized to the large centrioles present in the primary spermatocytes (Figure 1D). Taken together, these observations indicate that, like its *C. elegans* counterpart, DSpd-2 localizes both to the centrioles and to the PCM.

Interestingly, DSas-4, DSas-6, and Sak/Plk4 specifically localize to the proximal and distal ends of the centrioles in primary spermatocytes, and it has been proposed that this localization might be common to proteins involved in the duplication process [13]. In contrast, we observed DSpd-2 equally distributed along the length of the centrioles in these cells by using both our GFP fusion and our antibodies (Figure 1D; data not shown), suggesting that DSpd-2 might not contribute to centriole duplication in the same manner as do the other proteins.

### DSpd-2 Is Not Essential for Centriole Duplication

**Figure 2:**
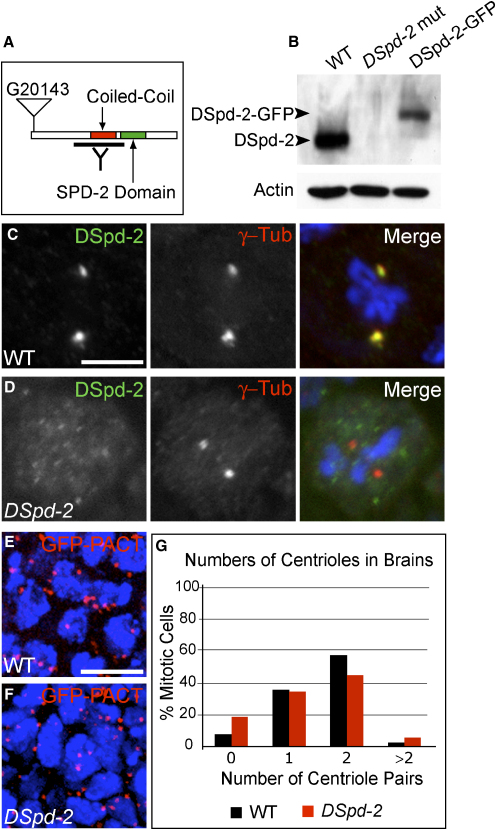
DSpd-2 Is Not Essential for Centriole Duplication in Cells of the *Drosophila* Brain (A) A schematic representation of the DSpd-2 protein and the position of the G20143 *P* element insertion. DSpd-2 is 1146 amino acids (aa) in length and was identified in the basis of the SPD-2 Domain (green) of ∼200 aa [1]. DSpd-2 also has an adjacent conserved coiled-coil region (red). The region against which the DSpd-2 antibody was raised is indicated (black line). (B) Western blot of protein extracts from third-instar larval brains, probed with DSpd-2 antibodies. Actin antibodies were used as a loading control. A band of the predicted size for DSpd-2 (125 kDa) is detected in WT but not *DSpd-2* mutant brains, indicating antibody specificity. A larger band (of ∼155 kDa) is observed in *DSpd-2* mutant brains expressing DSpd-2-GFP. (C and D) WT and *DSpd-2* third-instar larval neuroblasts stained for DSpd-2 (green), γ-tubulin (red), and DNA (blue). DSpd-2 protein is detectable at the centrosome of WT (C) but not mutant (D) neuroblasts. (E and F) Maximum-intensity projections of stacks taken through WT (E) and *DSpd-2* (F) whole-mount third-instar larval brains, expressing the centriole marker GFP-PACT (red). DNA is in blue. Large numbers of centrioles are observed in both WT and mutant brains. (G) A graph showing the percentage of mitotic (Phospho-Histone-H3 positive) cells with 0, 1, 2, or > 2 D-PLP-positive dots (centriole pairs). For WT cells, n = 546; for *DSpd-2* cells, n = 484. Cells were counted from at least six brains for both the WT and mutant. A dramatic reduction in centriole number is not observed in *DSpd-2* mutant brains compared to the WT. Scale bars represent 10 μm in (C) and (D) and 5 μm in (E) and (F).

To test whether DSpd-2 is essential for centriole duplication, we obtained a stock carrying a mutation in the *DSpd-2* gene. The G20143 mutant stock (see the Supplemental Experimental Procedures) is an enhanced and promoter (EP) line carrying a *P* element insertion directly after the initiating ATG codon of the *DSpd-2* gene (Figure 2A). On a western blot, DSpd-2 protein was not detected in *DSpd-2* mutant brains (Figure 2B), and quantitative western-blot analysis revealed that DSpd-2 protein levels were reduced by more than 90% (see the Supplemental Experimental Procedures). We also failed to detect any DSpd-2 at centrosomes or centrioles in *DSpd-2* homozygous mutant brain cells by immunofluorescence (Figures 2C and 2D). Thus, this *P* element insertion severely reduces the expression of the DSpd-2 protein. All of the phenotypes described below were rescued by the DSpd-2-GFP transgene (Figure 2B), demonstrating that the lack of DSpd-2 is responsible for these defects.

Previous studies have shown that flies lacking centrioles are viable but are severely uncoordinated because of a lack of cilia in their mechanosensory neurons [10]. However, *DSpd-2* mutant flies are viable and are not uncoordinated, suggesting that they possess both centrioles and cilia. Consistent with this observation, centrioles were detectable in both wild-type (WT) and *DSpd-2* mutant brains expressing the centriolar markers GFP-PACT, DSas-4-GFP, and Asl-GFP (Figures 2E and 2F; data not shown). Quantification of centriole numbers in fixed brain preparations revealed that, in contrast to mutants lacking the other centriole-duplication proteins DSas-4, DSas-6, and Sak/Plk-4 [10, 11, 13], there was no dramatic decrease in centriole numbers in *DSpd-2* mutant cells compared to the WT (Figure 2G). These results are consistent with the recent finding that depletion of *DSpd-2* in a genome-wide screen in *Drosophila* cultured cells did not affect centriole duplication [18].

To determine whether DSpd-2 is essential for centriole duplication in other tissues, we examined the large centrioles in the primary spermatocytes of the male germline. Strikingly, centriole numbers actually increased in the primary spermatocytes lacking DSpd-2 (Figure S1), suggesting that centrioles can over duplicate in the absence of DSpd-2 in some tissues. The presence of extra centrioles leads to the formation of multipolar spindles, resulting in severe meiotic defects and male sterility (data not shown). Taken together, these results suggest that DSpd-2 is not essential for centriole duplication in *Drosophila* somatic cells. Thus, SPD-2 is the first centriole-duplication factor from the *C. elegans* pathway that does not have an essential role in this process in *Drosophila*.

### Mitotic PCM Recruitment Is Inefficient in the Absence of DSpd-2

**Figure 3:**
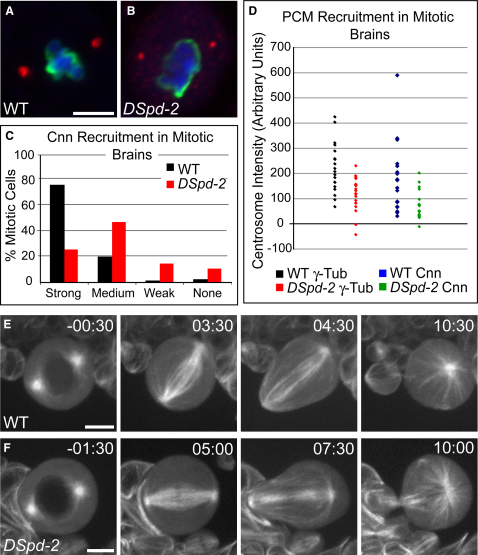
Centrosome Maturation Is Inefficient in the Absence of DSpd-2 (A and B) WT (A) and *DSpd-2* mutant (B) mitotic third-instar larval brains stained for Phospho-Histone-H3 (green), Cnn (red), and DNA (blue). WT cells strongly recruit Cnn to mitotic centrosomes, whereas Cnn recruitment in the *DSpd-2* mutant is clearly reduced. (C) A graph showing the percentage of mitotic (Phospho-Histone-H3 positive) cells with strong, medium, weak, or no Cnn staining at the centrosome. Cells were counted in at least six brains for each sample. For the WT, n = 526; for *DSpd-2*, n = 359. (D) A graph showing the centrosomal intensity of γ-tubulin (black and red) and Cnn (blue and green) staining of individual centrosomes in WT and mutant fixed mitotic third-instar larval neuroblasts. (E and F) Stills from movies of WT (E) and mutant (F) neuroblasts expressing GFP-α-tubulin (taken from Movies S2 and S3, respectively). Both WT and mutant cells drive the assembly of a robust mitotic spindle from their centrosomes (WT n = 47, *DSpd-2* n = 108; times relative to nuclear envelope breakdown [NEBD] are shown in top left). Scale bars represent 10 μm in (A) and (B) and 5 μm in (E) and (F).

In the *C. elegans* embryo, SPD-2 is essential for PCM recruitment to the centrioles, and it appears to be one of the earliest effectors in the recruitment pathway [1, 4]. To test whether DSpd-2 is also required for this process in flies, we assessed the recruitment of the PCM markers Cnn and γ-tubulin to mitotic centrosomes in WT and *DSpd-2* mutant third-instar larval brain cells. Both Cnn and γ-tubulin were still detectable on the majority of mitotic centrosomes in *DSpd-2* brains (Figures 3A–3C; data not shown), but they were present at significantly decreased levels compared to the WT (p < 0.01 and p < 0.001, respectively; Figure 3D). Thus, DSpd-2 appears not to be essential for PCM recruitment in mitotic larval brain cells, but is required to ensure the efficiency of this process.

We wondered whether the reduced levels of PCM at *DSpd-2* mutant centrosomes would affect the ability of the centrosomes to nucleate MTs and hence drive spindle formation. We found that the mitotic index in WT and *DSpd-2* mutant third-instar larval brain cells was very similar (2.6% ± 2.0, n > 10,000 cells for the WT; 2.7% ± 2.4, n > 13,000 cells for *DSpd-2* mutants), suggesting that mitosis occurs with normal timing in mutant cells. Moreover, a live analysis of the mitotic spindles in larval neuroblasts expressing GFP-α-tubulin revealed that the centrosomes in *DSpd-2* mutant neuroblasts nucleated robust astral MT arrays that participated in spindle formation in a manner indistinguishable from those in WT cells (Figures 3E and 3F, Movies S2 and S3). In addition, *DSpd-2* mutant neuroblasts invariably divided asymmetrically (n = 108), as was the case in WT neuroblasts (n = 47). Thus, in *DSpd-2* mutant third-instar larval brain cells, the reduced efficiency of PCM recruitment does not detectably perturb mitosis. This is in contrast to *C. elegans* embryos lacking SPD-2, in which PCM recruitment, MT nucleation, and mitosis are dramatically impaired [1, 4, 19].

### DSpd-2 Is Required for PCM Recruitment to the Sperm Centriole That Enters the Egg at Fertilization

Our data suggest that DSpd-2 is not essential for centriole duplication and has only a minor role in PCM recruitment in *Drosophila* somatic cells. Despite this fact, *DSpd-2* mutant females produced embryos that did not hatch as larvae. In fixed 0–4 hr collections of *DSpd-2* embryos mated with WT males (hereafter *DSpd-2* embryos), most embryos had been fertilized (>95% contained a sperm tail, n > 400) but were arrested very early in development.

**Figure 4:**
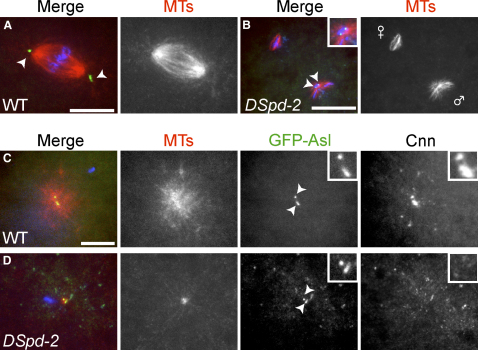
DSpd-2 Is Essential for PCM Recruitment and MT Nucleation from the Sperm Centriole WT or *DSpd-2* embryos expressing Asl-GFP (centriole marker) were stained to reveal the distribution of GFP, MTs, DNA, and Cnn. (A) The first mitotic spindle in a WT embryo. Shown are MTs (red), centrioles (green), and DNA (blue). A single centriole is visible at each spindle pole (arrowheads). (B) A *DSpd-2* mutant embryo at the equivalent stage (based on polar body morphology; not shown) as the WT embryo shown in (A); MTs (red), Centrioles (green), and DNA (blue) are shown. The male and female pronuclei (marked) have failed in pronuclear fusion. Two centrioles (arrowheads) are associated with the male pronucleus (see inset). (C and D) WT and *DSpd-2* sperm asters at anaphase or telophase of meiosis II. Shown are MTs (red), centrioles (green), and DNA (blue). WT embryos (C) have a large sperm aster associated with the centrioles, which by this time have clearly duplicated (arrowheads). Cnn protein is strongly recruited to these centrioles and the surrounding aster (see inset, far-right panel). In *DSpd-2* embryos (D), centriole duplication has still occurred (arrowheads), but the sperm aster is very small, and Cnn is absent from the centrioles (see inset, far-right panel). Scale bars represent 10 μm in (A), (C), and (D) and 20 μm in (B). The inset in (B) is a 7× magnification of the image. The insets in (C) and (D) are 3× magnifications of the image.

To determine the nature of the defect in *DSpd-2* embryos, we fixed 0–15 min collections of embryos and stained them to visualize MTs, PCM (Cnn), and centrioles (Asl-GFP). The first mitotic spindle was frequently observed in 0–15 min collections of WT embryos (Figure 4A), but it was never observed in *DSpd-2* embryos (∼900 embryos scored), suggesting that mutant embryos arrest before the first mitosis. In many mutant embryos, it was clear that pronuclear migration had failed, and we often observed male and female pronuclei that remained spatially separated within the embryo (Figure 4B). This was never observed in WT embryos of a similar stage (as determined by the morphology of the polar bodies; data not shown). Thus, it appears that *DSpd-2* mutant embryos are unable to develop because of a failure in pronuclear fusion.

We next analyzed whether there were defects in female meiosis that might explain the early arrest of the *DSpd-2* embryos. We found that female meiosis proceeded normally in *DSpd-2* embryos, generating a female pronucleus that was positioned appropriately for subsequent capture by the sperm aster. We did, however, observe defects in the recruitment of Cnn to the shared central pole of the meiosis II spindles in *DSpd-2* embryos (Figure S2). This observation suggests that DSpd-2 can have a role in recruiting PCM proteins to MT organizing centers (MTOCs) that do not contain centrioles.

To test whether the mutant embryos fail in pronuclear fusion because of a defect in sperm aster assembly, we examined the distribution of PCM and MTs around the sperm centriole that enters the egg at fertilization. In the majority of WT embryos in metaphase or later stages of meiosis II, large amounts of Cnn (8/9 embryos) and MTs (7/9 embryos) were recruited around the sperm centriole (Figure 4C). In contrast, in *DSpd-2* embryos at the same stage, sperm centrioles were rarely associated with Cnn (1/6 embryos) or MTs (1/6 embryos) (Figure 4D). Thus, in contrast to somatic cells, DSpd-2 has an essential role in recruiting the PCM to the sperm centriole: In its absence, the sperm centrosome does not organize a robust array of astral MTs, pronuclear fusion fails, and mutant embryos arrest prior to the first mitotic division.

These results suggest that DSpd-2 has a particularly important role in PCM recruitment to the sperm centriole. We therefore wondered whether DSpd-2 might also be essential for centriole duplication specifically during the first centriole-duplication event after fertilization. We observed, however, that in all late meiosis II embryos in which we could detect the centrioles, a newly duplicated centriole could be distinguished in both WT (Figure 4C; n = 6) and *DSpd-2* (Figure 4D; n = 3) embryos. This suggests that the single sperm-derived centriole is capable of undergoing the first duplication event in the absence of maternally supplied DSpd-2 protein, although the interpretation of this result is not straightforward (discussed below).

### A Specialized Role for SPD-2 Proteins in Recruitment of Proteins to the Sperm Centrioles?

Our data indicate that the *Drosophila* ortholog of *C. elegans* SPD-2 is not essential for centriole duplication and has only a minor role in PCM recruitment in somatic cells. This might suggest that the function of SPD-2 has diverged between worms and flies. We note, however, that the essential role of *C. elegans* SPD-2 in centriole duplication and PCM recruitment has, to date, only been demonstrated in the embryo, during the first events after fertilization [1, 4, 19]. Our data show that DSpd-2 plays a particularly important role in recruiting the PCM to the centriole that enters the fly embryo at fertilization. Thus, it is possible that this family of proteins has a conserved function, which is only absolutely essential when the sperm centriole(s) first enter the fertilized egg.

Why would SPD-2 proteins be essential for PCM recruitment to the sperm centriole but not to the centrioles in somatic cells? One possibility is that SPD-2 proteins have a specialized role in facilitating the de novo recruitment of PCM proteins from maternal stores to the “naked” centriole(s) that enter the oocyte at fertilisation. This is a unique situation, because in all subsequent rounds of mitotic PCM recruitment, the centrioles are already associated with at least a small amount of PCM that was present on the centriole during interphase. Hence, SPD-2 proteins might not be essential for PCM recruitment once the PCM has initially been loaded onto the sperm centrioles. It remains possible, however, that SPD-2 proteins are also essential for PCM recruitment to the centrioles during subsequent embryonic cycles. Unfortunately, we cannot test this possibility because *DSpd-2* embryos arrest prior to the first mitotic division.

As is the case for PCM recruitment, there are currently no data suggesting that SPD-2 is essential for centriole duplication in worm somatic cells. Indeed, the results of a recent candidate-based siRNAi screen in human tissue culture cells suggested that human SPD-2 (Cep192) might not be essential for centriole duplication in somatic cells [20]. It therefore remains possible that SPD-2 proteins could be specifically required for the initial duplication of the fertilizing sperm centriole. At a first glance, our data appear to contradict this hypothesis because we show that the first round of centriole duplication can occur in *Drosophila* embryos lacking DSpd-2. It remains possible, however, that the initial events of centriole duplication have already occurred in the sperm prior to fertilization, and so are not dependent on the maternally supplied pool of DSpd-2. It is not possible to test this hypothesis directly by the fertilization of *DSpd-2* embryos with *DSpd-2* mutant sperm because these sperm are immotile.

How could SPD-2 proteins coordinate these two important centrosome-cycle events—centriole duplication and centrosome maturation? We propose that SPD-2 could act as a general protein-recruitment factor, which is only absolutely essential for the recruitment of centriole-duplication factors and PCM proteins to the sperm centriole(s) after fertilization. More work will elucidate whether SPD-2 proteins are also dispensable for these processes in the somatic cells of other species, or whether the functions of worm and fly SPD-2 proteins have diverged. In either case, our data highlight the importance of studying the roles of proteins implicated in the centrosome cycle in a range of organisms and cellular contexts.

## Supplemental Data

Document S1. Experimental Procedures and Two Figures

Movie S1. Movie of Syncitial Stage Embryo Expressing DSpd-2-GFP This embryo is in telophase at the start of the movie, and the duplicated centrosomes are just separating. DSpd-2-GFP is present on the centrosomes throughout the embryonic cell cycle. An increase in the level of DSpd-2 is seen as the embryo goes into mitosis and the centrosomes mature, indicating that DSpd-2-GFP is a PCM component. Each frame represents 1 s and the movie runs at 45 fps.

Movie S2. WT Neuroblast Undergoing an Asymmetric Mitotic Division The cell is already in mitosis at the start of the movie, just prior to nuclear envelope breakdown (NEBD). Both centrosomes nucleate robust astral MTs, which drive spindle formation. The spindles also have robust astral MTs, and the cell divides asymmetrically. Each frame represents 30 s and the movie runs at 10 fps.

Movie S3. *DSpd-2* Mutant Neuroblast Undergoing an Asymmetric Mitotic Division This cell is already in mitosis at the start of the movie, just prior to NEBD. As in WT neuroblasts, both centrosomes nucleate robust astral MTs, which drive spindle formation. The spindles also have robust astral MTs, and the cell divides asymmetrically. Each frame represents 30 s and the movies runs at 10 fps.
