# Supplementary material for: Drosophila Spd-2 Recruits PCM to the Sperm Centriole, but Is Dispensable for Centriole Duplication
Source: Curr Biol. 2007 Oct 23;17(20):1759–64. doi: 10.1016/j.cub.2007.08.065 (PMC2045633; doi:10.1016/j.cub.2007.08.065)
Supplement: Document S1. Experimental Procedures and Two Figures [file mmc1.pdf]

Supplemental Data

Drosophila Spd-2 Recruits PCM to the Sperm Centriole, but Is Dispensable for Centriole Duplication

Carly I. Dix and Jordan W. Raff

Supplemental Experimental Procedures

Generation of DSpd-2 Antibodies

DNA encoding amino acids 352–758 of the DSpd-2 protein (CG17286) was amplified by polymerase chain reaction (PCR). This was subcloned into the pMAL expression vector (New England Biolabs [Ipswich, MA]) and an MBP-fusion protein was expressed in *E. coli* and purified as described previously [S1]. Antisera were raised in two different rabbits by Eurogentec (Seraing, Belgium). Antibodies were affinity purified and stored as described previously [S2].

Generation of the DSpd-2-GFP Fusion Protein and Transgenic Lines

The complete coding sequence of the *DSpd-2* gene was amplified from genomic DNA with primers to allow subsequent Gateway cloning (Invitrogen) (details available on request). The resulting fragment was introduced into the pDONR-Zeo vector and then recombined into a Ubq-GFP plasmid that would drive the expression of a DSpd-2-GFP fusion protein at moderate levels in all cells [S3]. Transgenic lines were generated by standard *P* element-mediated transformation.

Fly Stocks

In all experiments, Oregon R flies were used as wild-type controls. We obtained the mutant stock $DSpd\text{-}2^{G02143}$ from the GeniSys database of EP lines (GenExel [South Korea]). The mutant chromosome contains a *P* element insertion within the first exon of the *DSpd-2* gene, just one nucleotide after the ATG. The maternal-effect lethality and male sterility observed in mutant flies is also observed when the mutant chromosome is crossed to the deficiency Df(3L)st-j7 (Bloomington stock #5416).

The Asl-GFP (C. Gonzalez, personal communication), DSas-4-GFP [S3], GFP-PACT [S4], GFP-DSas6 [S3], and mRFP-PACT [S3] transgenic lines all contain GFP or red fluorescent protein (RFP) fusions driven by the pUbq promoter, and so are expressed in all tissues. To visualize spindles in larval neuroblasts, we used a transgenic line expressing GFP-α-tubulin line under the control of a UAS promoter, recombined to the MZ1407 driver [S5].

Live Analysis of GFP Fusion Proteins in Larval Neuroblasts and Syncytial Embryos

Embryos expressing DSpd-2-GFP were aligned and subsequently observed on a Perkin Elmer spinning-disc confocal system as described previously [S6]. Live analysis of third-instar larval neuroblasts was performed as described previously [S5] but with a Perkin Elmer ERS spinning-disc confocal system [S6]. We imaged the entire depth of the cell by taking a Z stack of images spaced 0.5 mm apart that spanned this entire distance. Z stacks were taken at 30 s intervals. All images shown are maximum-intensity projections, and all images were processed with Volocity (Improvision) software. All control and experimental images were adjusted with the same procedures, which were applied to the whole image.

Fixed Analysis of Larval Testes, Larval Brains, Adult Testes, and Early Embryos

Third-instar larval brains and testes were dissected, fixed, stained, and analyzed as described previously [S4]. We obtained the mitotic index of fixed cells by staining fixed preparations of larval neuroblasts with Hoechst and Phospho-Histone-H3 antibodies and counting the ratio of Phospho-Histone-H3-positive to -negative cells. This was done in an automated fashion with Metamorph to count all nuclei, with manual correction and scoring of mitotic cells. Adult testes expressing RFP-PACT were dissected from both WT and *DSpd-2* mutant testes. Fixed samples of intact 16 cell cysts of primary spermatocytes were prepared from adults with the method described previously [S7], except that in all steps where present, acetic acid was omitted so that the RFP-PACT signal could be retained. Cysts were obtained from four individual flies for the WT and three individual flies for mutants. Early embryos were fixed as described previously [S2]. We visualized centrioles in early embryos by staining embryos expressing Asl-GFP with an anti-GFP antiserum.

Antibodies

In this study, the following antibodies were used: rabbit anti-DSpd-2 (described above); rabbit anti-D-PLP [S4]; GTU88 mouse monoclonal anti-γ-tubulin (Sigma); mouse anti-Phospho-Histone-H3 (AbCam, UK); rabbit anti-centrosomin [S3]; guinea pig anti-centrosomin (E.P. Lucas, personal communication); mouse anti-actin monoclonal C4 (ICN Biomedicals [Costa Mesa, CA]); rabbit anti-GFP antiserum A6455 (Molecular Probes, Invitrogen); DM1α mouse monoclonal anti-α-tubulin (Sigma); and GTU88*, a batch of the GTU88 monoclonal antibody (Sigma) that crossreacts with centrioles in *Drosophila* [S4]. Affinity-purified antibodies were used at 1–2 μg/ml in immunofluorescence and immunoblotting experiments, and all sera or commercial antibodies were used at a 1:500 dilution. Appropriate HRP-, Alexa 488-, Cy3- and Cy5-conjugated secondary antibodies were obtained from Molecular Probes, Jackson Laboratories, or Amersham Biosciences.

Electron Microscopy

Testes from WT and *DSpd-2* adult flies were dissected in phosphate-buffered saline (PBS) and washed in 0.9% NaCl. The testes were fixed in 2% glutaraldehyde in 0.1M PIPES (pH 7.4) for 3 hr at 4°C and then washed twice in 0.1M PIPES (pH 7.4). Samples were then processed for electron microscopy (EM) as described previously [S4]. Sections taken from four WT and five mutant testes were analyzed.

Electrophoresis and Immunoblotting

For Western blotting, ten WT and ten *DSpd-2* brains were dissected in PBS and homogenized in sodium dodecyl sulfate (SDS) sample buffer. We separated the proteins on a 4%–12% gradient precast acrylamide gel NuPAGE (Invitrogen) and then transferred them by electroblotting to a Hybond-P membrane (Amersham Biosciences). Western blotting was performed as described previously [S2]. So that the sensitivity of our DSpd-2 antibodies on a western blot could be determined, WT embryo extract (20 embryos, 0–4 hr old) was serially diluted with PBS, giving a range of dilutions (100%, 50%, 20%, 10%, 1%, and 0.1%), and was probed with our DSpd-2 antibody. We found that we could reproducibly detect DSpd-2 in extract diluted to just 10% of WT levels. Because we are unable to detect DSpd-2 protein in our *DSpd-2* mutant embryo extract, we estimate that the levels of DSpd-2 must therefore be depleted by greater than 90%.

Quantitation of Centrosomal Fluorescence

The centrosomal fluorescence of γ-tubulin and Cnn staining at mitotic centrosomes was measured in fixed samples of both WT and *DSpd-2* third-instar larval brains with a Zeiss Axioskop II microscope with a CoolSnapHQ camera (Photometrics) and Metamorph software (Molecular Devices). Fluorescence intensity was measured from maximum-intensity projections of image stacks spanning 1.5 μm, taken at 0.3 μm intervals. The mean fluorescence intensities were measured in a small area that was manually positioned around the centrosomes in these projections. The centrosomal intensity was measured for 20 individual centrosomes for each experiment, each taken from different cells, and the mean pixel intensity of the background was subtracted from this value. Cells were analyzed from four different samples. The significance of the difference between the mean centrosomal intensities of each marker in WT and *DSpd-2* mutant cells was tested with a two-tailed Student's t test.

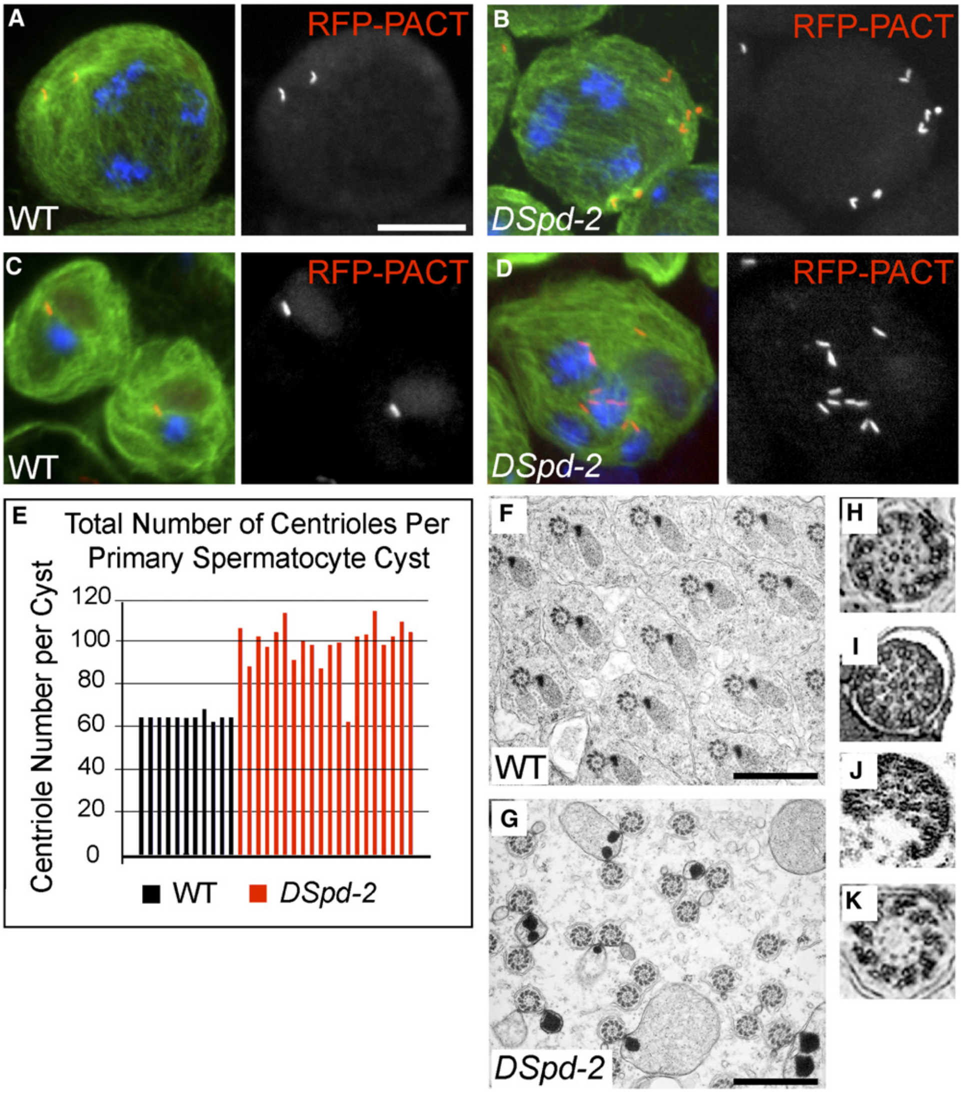

Figure S1. *DSpd-2* Mutant Spermatocytes Have Increased Numbers of Centrioles

Drosophila spermatogenesis begins at the apical tip of the testis, where a germline stem cell (GSC) divides asymmetrically to generate another GSC and a gonialblast. The gonialblast undergoes four rounds of mitotic divisions to form a cyst of 16 primary spermatocytes [S8]. The centrioles in the primary spermatocytes enlarge and become structurally more elaborate than those in other *Drosophila* cells [S9].

(A–D) WT (A and C) and *DSpd-2* mutant (B and D) spermatocytes expressing RFP-PACT (red), stained for MTs (green) and DNA (blue). Meiotic (prophase I) WT spermatocytes contain two enlarged V shaped centriole pairs (A). *DSpd-2* mutant spermatocytes contain extra structures with the appearance of centrioles (B). The majority of these extra centrioles are present as V shaped pairs, whereas a few are present as single centriole barrels. In spermatogenesis, after meiotic onset, the four centrioles present in each mature spermatid do not duplicate again. Thus, after two rounds of meiosis, each of the 64 spermatids present within a cyst contains a single centriole, which subsequently matures to a basal body and templates the sperm flagellum. Each WT onion stage (post-meiosis II) spermatid has a single nucleus and a single centriole (basal body) (C). Equivalent stage *DSpd-2* spermatids frequently have extra centrioles and have abnormal numbers of nuclei that are often unequal size, indicating earlier problems in cytokinesis and chromosome segregation during meiosis (D).

(E) A graph showing the total number of single centrioles observed in individual 16 cell primary spermatocyte cysts in WT and *DSpd-2* testes (WT n = 11 cysts; *DSpd-2* n = 20 cysts). In WT testes, we found that all cysts contained 16 cells, and in the vast majority of cysts, we observed the expected 64 centrioles (organized as two pairs per primary spermatocyte). In *DSpd-2* mutant testes, we consistently observed 16 primary spermatocytes per cyst, but the number of centriole per cyst was almost always greater than 64. Because 16 cell cysts were always observed, the extra centrioles present have not arisen because of defects in cytokinesis.

(F–K) EM analysis of the flagellar axoneme in WT (F and H), and *DSpd-2* mutant (G and I–K) adult testes. The WT tissue has a uniform and stereotypical layout (F). *DSpd-2* mutant tissue is very disorganized, probably as a result of spindle multipolarity during meiosis I and II (G). WT axonemes have the characteristic 9 + 2 structure in cross-section (H). Most *DSpd-2* axonemes also have a normal 9 + 2 structure, although a small proportion has structural defects (I). A *DSpd-2* mutant axoneme with incomplete structure (4%; n = 334) (J) and a *DSpd-2* mutant axoneme with missing central pair (0.3%; n = 334) (K) are shown. Therefore, the majority of axonemes present are structurally normal and have been correctly templated in *DSpd-2* mutants.

Scale bars represent 10 μm in (A)–(D) and 1 μm in (F) and (G); the panel width is 300 nm in (H)–(K).

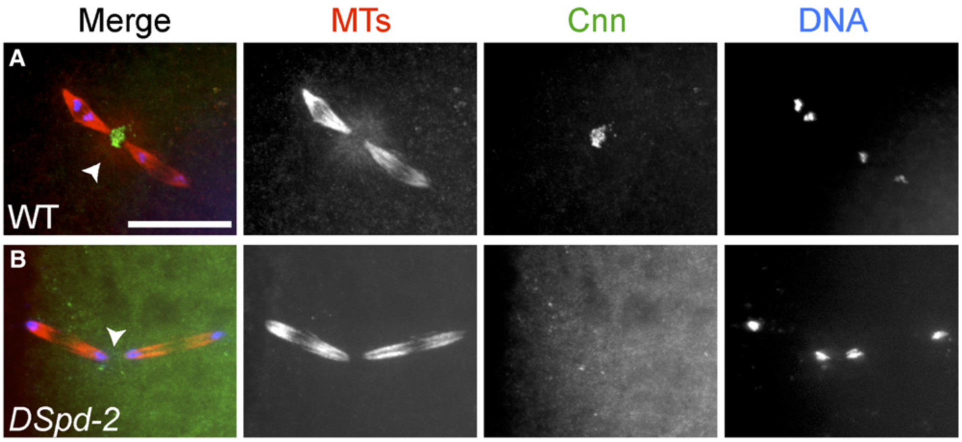

Figure S2. DSpd-2 Is Essential for Cnn Recruitment and MT Nucleation at the Central Pole of the Female Meiosis II Spindle

In *Drosophila,* the female meiosis II spindle is acentrosomal and is orientated perpendicular to the embryo cortex [S10]. This stereotypical arrangement ensures that the innermost haploid meiotic product (the future female pronucleus) is positioned appropriately for capture by the large aster of MTs nucleated from the sperm centrosome [S11]. The meiotic spindle in WT embryos from metaphase onward of meiosis II has a prominent central pole (arrowhead), to which Cnn localizes and from which MTs are nucleated, as described previously [S12, S13] (A). (B) Equivalent *DSpd-2* mutant meiotic spindles almost always lacked Cnn and central astral MTs at this region (arrowhead) (B). This suggests that DSpd-2 might have some role in recruiting PCM proteins to MTOCs that do not contain centrioles. Shown are MTs (red), Cnn (green), and DNA (blue). The scale bar represents 20 μm.

Supplemental References

S1. Gergely, F., Kidd, D., Jeffers, K., Wakefield, J.G., and Raff, J.W. (2000). D-TACC: A novel centrosomal protein required for normal spindle function in the early Drosophila embryo. EMBO J. *19*, 241–252.

S2. Huang, J., and Raff, J.W. (1999). The disappearance of cyclin B at the end of mitosis is regulated spatially in Drosophila cells. EMBO J. *18*, 2184–2195.

S3. Peel, N., Stevens, N.R., Basto, R., and Raff, J.W. (2007). Overexpressing centriole-replication proteins in vivo induces centriole overduplication and de novo formation. Curr. Biol. *17*, 834–843.

S4. Martinez-Campos, M., Basto, R., Baker, J., Kernan, M., and Raff, J.W. (2004). The Drosophila pericentrin-like protein is essential for cilia/flagella function, but appears to be dispensable for mitosis. J. Cell Biol. *165*, 673–683.

S5. Basto, R., Lau, J., Vinogradova, T., Gardiol, A., Woods, C.G., Khodjakov, A., and Raff, J.W. (2006). Flies without centrioles. Cell *125*, 1375–1386.

S6. Barros, T.P., Kinoshita, K., Hyman, A.A., and Raff, J.W. (2005). Aurora A activates D-TACC-Msps complexes exclusively at centrosomes to stabilize centrosomal microtubules. J. Cell Biol. *170*, 1039–1046.

S7. Bonaccorsi, S., Giansanti, M.G., Cenci, G., and Gatti, M. (2000). Cytological analysis of spermatocyte growth and male meiosis in Drosophila melanogaster. In Drosophila Protocols, W. Sullivan, M. Ashburner, and R.S. Hawley, eds. (Cold Spring Harbor, New York: Cold Spring Habor Laboratory Press), pp. 87–109.

S8. Fuller, M. (1993). Spermatogenesis. In The Development of Drosophila melanogaster, M. Bate and A. Martinez-Arias, eds. (Cold Spring Harbor, New York: Cold Spring Harbor Laboratory Press), pp. 71–147.

S9. Gonzalez, C., Tavosanis, G., and Mollinari, C. (1998). Centrosomes and microtubule organisation during Drosophila development. J. Cell Sci. *111*, 2697–2706.

S10. Endow, S.A., and Komma, D.J. (1997). Spindle dynamics during meiosis in Drosophila oocytes. J. Cell Biol. *137*, 1321–1336.

S11. Foe, V.E., Odell, G.M., and Edgar, B.A. (1993). Mitosis and morphogenesis in the Drosophila embryo. In The Development of Drosophila melanogaster, M. Bate and A. Martinez-Arias, eds. (Cold Spring Harbor, New York: Cold Spring Harbor Laboratory Press), pp. 149–300.

S12. Llamazares, S., Tavosanis, G., and Gonzalez, C. (1999). Cytological characterisation of the mutant phenotypes produced during early embryogenesis by null and loss-of-function alleles of the gammaTub37C gene in Drosophila. J. Cell Sci. *112*, 659–667.

S13. Riparbelli, M.G., and Callaini, G. (2005). The meiotic spindle of the Drosophila oocyte: The role of centrosomin and the central aster. J. Cell Sci. *118*, 2827–2836.
